# 

**Published:** 1985-04

**Authors:** 


APRIL 1985

VOLUME 100 (ii)

NUMBER 374

Published Quarterly

Annual Subscription £10 Post Free

### BRISTOL MEDICO-CHIRURGICAL JOURNAL

Contents

31. Editorial

Established 1883

### BRISTOL MEDICO-CHIRURGICAL JOURNAL

Incorporating the Medical Journal of the South West

*Scire est nescire, nisi id me scire alius sciret*—
Lucilius 180–102 BC

**55.** **Wessex Branch of the Association of Clinical Pathologists**
**Abstracts of Meeting in Bristol, November 1984**

**58.** **South West Orthopaedic Club**
**Abstracts of Meeting in Exeter, November 1984**

**60.** **University of Bristol**

**Examination Results**

Printed in Great Britain by John Wright & Sons (Printing) Ltd, at The Stonebridge Press, Bristol

### Editorial Committee

**EDITOR**
*Mr. M. G. Wilson*
*40 Coombe Lane, Bristol BS9 2BJ*

MEMBERS
*Dr. M. B. Lennard*
*Dr. D. W. Wright*
*The Hon. Treasurer of the Society*
*The Hon. Secretary of the Society*

SECRETARY
*Mr. B. P. Jones, The Library, Medical School, University Walk, Bristol BS8 1TD*

### Correspondents

*Dr. J. D. Davies*
*Dr. J. Jancar*
*Dr. H. G. Mather*
*Professor G. M. Stirrat*
*Dr. J. L. G. Thomson*
*Dr. M. J. Whitfield*
*Professor R. C. N. Williamson*
*Dr. D. W. Wright*

### Bristol Medico-Chirurgical Society

PRESIDENT

*Dr I. S. Bailey*

PRESIDENT ELECT
*Dr J. Jancar*

HONORARY SECRETARY
*Dr. H. M. Norman*
*Hillside, Vicarage Lane, Barrow Gurney*

HONORARY TREASURER
*Dr. J. B. Penry.*
*Southmead Hospital, Bristol BS10 5NB*

The Society was founded in 1874. In 1883 publication of the Journal began and has continued quarterly ever since then. During the years 1949 to 1962 it appeared under the title of 'The Medical Journal of the South West'.

### Notice to Contributors

*The Journal is especially concerned to provide a place for the recording of medical thought, interests and practice in and around Bristol. It therefore welcomes articles that, as well as being of immediate interest to members, will document the local and contemporary medical scene for our successors.*

*Original articles are preferred in the form proposed by the International Committee of Medical Editors (The Vancouver System) and published as an article in the British Medical Journal in 1979 (Br. Med. J.* **1***, 532–535). This has been reprinted as a pamphlet and may be obtained from the Editor of the B.M.J., B.M.A. House, Tavistock Square, London WC1H 9JR (enclose 50p).*

*Also welcome are letters to the Editor, Book Reviews, news of important appointments and events, etc.*

### University of Bristol Faculty of Medicine

### THE DEGREE OF MASTER OF SCIENCE IN MEDICAL SCIENCES
**January 1985**

Muftah Bashir ELKOM
Fahema El-Neffati OSMAN

### THE DEGREE OF DOCTOR OF PHILOSOPHY
**January 1985**

Peter Nicholas BURNS
Eleni Nikolaou DOTSIKA
Mark Johnathan Ross HAMILTON
Robert JENKINS

### THE DEGREE OF DOCTOR OF MEDICINE
**January 1985**

Nicholas David CARR

### FINAL EXAMINATION FOR THE DEGREES OF M.B., Ch.B.
**December 1984**

PASS

Carolyn Grace ABBOTT
Manjit Kaur BHOGAL
Fiona Jane BLUNDELL
Lance Gregory HERRON
Enid JONES
Roger Michael LANE
Jonathan Michael LEWIS
Lorraine May LOVEDEN
Vidhu MAYOR
Alex MORAN

